# Development and Usability Testing of a Web-Based and Therapist-Assisted Coping Skills Program for Managing Psychosocial Problems in Individuals With Hand and Upper Limb Injuries: Mixed Methods Study

**DOI:** 10.2196/17088

**Published:** 2020-05-06

**Authors:** Folarin Omoniyi Babatunde, Joy MacDermid, Ruby Grewal, Luciana Macedo, Mike Szekeres

**Affiliations:** 1 School of Rehabilitation Science Institute of Applied Health Science McMaster University Hamilton, ON Canada; 2 Roth McFarlane Hand and Upper Limb Centre St. Joseph’s Health Care London Western University London, ON Canada; 3 Department of Physical Therapy Western University London, ON Canada; 4 Schulich School of Medicine and Dentistry Western University London, ON Canada

**Keywords:** usability testing, upper extremities, psychosocial, internet, coping skills

## Abstract

**Background:**

Ineffective coping has been linked to prolonged pain, distress, anxiety, and depression after a hand and upper limb injury. Evidence shows that interventions based on cognitive behavioral therapy (CBT) may be effective in improving treatment outcomes, but traditional psychological interventions are resource intensive and unrealistic in busy hand therapy practices. Developing web-based, evidence-based psychological interventions specifically for hand therapy may be feasible in clinical practice and at home with reduced training and travel costs. Hand Therapy Online Coping Skills (HOCOS) is a program developed to supplement traditional hand therapy with therapist-assisted coping skills training based on principles from CBT and the Technology Acceptance Model.

**Objective:**

This study aimed to describe the development and assess the usability of HOCOS to support hand therapists in the management of psychosocial problems.

**Methods:**

The ADDIE model (Analysis, Design, Development, Implementation, and Evaluation) of system design was applied to create HOCOS. The usability testing of HOCOS involved a 2-stage process. In the first step, heuristic testing with information and communications technology (ICT) experts was completed using two sets of heuristics: Monkman heuristics and the Health Literacy Online (HLO) checklist. The second step involved user testing with hand therapists performing a series of online and face-to-face activities, completing 12 tasks on the website using the think-aloud protocol, completing the system usability scale (SUS) questionnaire, and a semistructured feedback interview in 2 iterative cycles. Descriptive statistics and content analyses were used to organize the data.

**Results:**

In total, 4 ICT experts and 12 therapists completed usability testing. The heuristic evaluation revealed 15 of 35 violations on the HLO checklist and 5 of 11 violations on the Monkman heuristics. Initially, hand therapists found 5 tasks to be difficult but were able to complete all 12 tasks after the second cycle of testing. The cognitive interview findings were organized into 6 themes: task performance, navigation, design esthetics, content, functionality and features, and desire for future use. Usability issues identified were addressed in two iterative cycles. There was good agreement on all items of the SUS. Overall, therapists found that HOCOS was a detailed and helpful learning resource for therapists and patients.

**Conclusions:**

We describe the development and usability testing of HOCOS; a new web-based psychosocial intervention for individuals with a hand and upper limb injuries. HOCOS targets psychosocial problems linked to prolonged pain and disability by increasing access to therapist-guided coping skills training. We actively involved target users in the development and usability evaluation of the website. The final website was modified to meet the needs and preferences of the participants.

## Introduction

### Background

Hand and upper limb injuries are some of the most common injuries in orthopedic settings [1,2], and approximately 11% to 20% of emergency department visits are because of hand and upper limb injuries [3,4]. In addition to pathophysiology, psychosocial factors can predict disability in individuals with hand and upper limb injuries [5,6]. These injuries have been shown to impact employment, body image [7], relationships [8], and functional abilities [9-11] negatively.

Most studies conducted in hand therapy have focused on maximizing physical recovery and adjustments with regard to medical or occupational therapy procedures [12-15]. Interventions such as joint protection [16], exercise therapy [17], mobilization [18], and modalities [19] in hand therapy have well-established benefits for pain and function. However, they do not directly target psychosocial factors that contribute to patient morbidity [20]. Several studies have established the mediating effect of psychological distress on hand and upper limb pain and disability [21-24] based on the far-reaching impact of psychosocial problems on pain and disability, and patient expectations after hand and upper limb injuries, a greater understanding of how to facilitate psychosocial adjustments is warranted [2,25]. Psychological interventions such as cognitive behavioral therapy (CBT) interventions have been shown to yield long-term [26] improvements in pain, daily function, quality of life, and overall mental health compared with active treatments alone for several musculoskeletal (MSK) problems [27,28] including knee pain [29], low back pain, [30,31] fibromyalgia, [32] preoperative spine [33] and post total joint surgery [34]. CBT is also cost-effective [35] and cost neutral when considering the overall health care sector and labor market perspective [33], with reduced health care utilization at the 5-year follow-up [26]. CBT has also been shown to be effective in improving adherence to exercise [36]. CBT techniques such as graded activity can be integrated into traditional physiotherapy [37,38].

In hand therapy, CBT may be efficient treatment to improve pain and distress by increasing adjustment to hand injury in relation to illness perception and coping strategies [2,39-41]. Unfortunately, traditional CBT is resource intensive and not feasible to implement in busy hand therapy practices because of prolonged face-to-face encounters and associated cost implications [42]. Web-based CBT is a potential emerging tool with modern interactive and communicative technologies for use in rural and urban areas, across languages and cultures, and on a global scale [43]. Web-based CBT has been shown to be effective for reducing catastrophization and improving the attitudes of patients with MSK conditions to exercise therapy [44]. Current evidence supports the feasible and efficacious delivery of web-based CBT using nontraditional health professionals such as physiotherapists (PTs) and occupational therapists (OTs) [45], with reduced time commitment and treatment costs, and positive self-reported changes in the PTs’ attitudes, confidence, and practice [46-48]. Therapist competence and therapeutic alliance are crucial factors influencing CBT [49]. Therapist competency can be developed online [50], and therapeutic alliance required for CBT to be effective does not diminish with the web-based delivery of CBT [51].

Hagemen et al [52] reported that almost 50% of outpatients presenting to hand surgery clinics investigated their symptoms online, which increases the potential to deliver evidence-based pain management and coping skills for HULI online. Further studies on the use of psychosocial interventions in HULI have the potential to convince payers to fund psychotherapy treatments, generate enthusiasm to include psychosocial treatments in educational curriculums, and advance incorporation of evidence-based psychosocial treatments in hand therapy recommendations for psychosocial problems [53]. In view of the evidence showing evidence-based CBT can be delivered via the internet and feasible to implement during wait times for hand therapy or in home-settings and reduce the costs associated with training providers and fewer hospital visits. From the foregoing, online evidence-based CBT is feasible to implement during wait times for hand therapy, is easy to use in home settings, and reduces costs associated with training providers and leads to fewer hospital visits. To meet the needs of patients with hand and upper limb injuries at risk of prolonged pain and disability because of psychosocial treatments, we decided to develop an intervention that incorporates evidence from CBT in orthopedic practice.

### Hand Therapy Online Coping Skills Program

Hand Therapy Online Coping Skills (HOCOS) is an evidence-based and theory-based psychosocial coping skills program based on CBT principles [37] and the Technology Acceptance Model [54]. HOCOS was developed by Folarin Babatunde (PT) during his doctoral studies at McMaster University In collaboration with a team of PTs (JM and LM), OT (MS), hand therapists (JM and MS) and an orthopedic surgeon (RG). HOCOS involves five ‘hand therapist-guided’ modules. It is a multi-component, interactive online-based program consisting of hand and upper-limb specific information covering pain education and training in coping skills (activity-rest cycling, pleasant activity scheduling, problem-solving, identifying and challenging negative thoughts, relaxation response and their applications) to daily life for adults with hand and upper limb injuries (Table 1). Asynchronous learning was facilitated using PowerPoint (Microsoft Office) presentations, audio files, workbooks, and downloadable PDF (Adobe) and Word (Microsoft Office) files. Links to evidence-based external educational resources were included to reinforce learning. The program was designed to supplement traditional hand therapy with coping skills training. The design and development of HOCOS were guided by the 5 steps of the Analysis, Design, Development, Implementation, and Evaluation from the ADDIE model [55,56]. The structure and specific ingredients of HOCOS were based on the following recommendations suggested by Bennell et al [57]: (1) impact of psychosocial factors on pain and disability in hand injuries, (2) evidence base for the effects of CBT on MSK conditions, (3) the importance of incorporating the management of hand injuries into a biopsychosocial framework, and (4) practical issues related to the delivery of the intervention.

**Table 1:** Outline of hand therapy online coping skills session contents.

Session	Projected duration	Outline of content
1	1 week	Logging in and account set up using provided password Completing battery of questionnaires (demographic information, self-report of hand pain and function, psychosocial factors, and assessment) Information provided about the psychosocial aspects of prolonged pain Introduction to the module contents Introduction to SMART (Specific, Measurable, Achievable, Realistic or Relevant and Timed) goals and using the program calendar to plan activities Providing information on contacts for technical difficulties
2	2 weeks	Module 1: introduction to pain (meaning, definition, and impact on recovery) This module teaches concepts from therapeutic neuroscience education using stories and metaphors Promote interest in exercise and physical activity
3	2-4 weeks	Module 2: introduction to the cognitive model Encourages users to identify and rate their moods Encourages users to reflect on their thinking style and identify patterns Encourages users to follow the guidelines for completing a thought record
4	2 weeks	Module 3: introduction to activity management principles Encourages users to pace activities to avoid boom and bust situations Encourages users to review their day planner and spot patterns of overactivity and underactivity Encourages users to focus on activities that have high mastery and pleasure value
5	2 weeks	Module 4: introduction to problems limiting recovery and how to solve them Encourages users to consider barriers to coping exercises and reflect on how to overcome those barriers Discussion on challenges to exercise and physical activity adherence and steps to regain control
6	2 weeks	Module 5: introduction to stress management, relaxation response, and sleep training Encourages users to reflect on the cycle of stress, muscle tension, and pain Encourages users to practice and adopt 1 or 2 relaxation techniques to their day plan Encourages users to include downtime in their daily plan
7	Posttraining (single or multiple modules)	This session focuses on how to continue recommended activities after completing the program Users are encouraged to continue to access the resources on the website if necessary Clinicians are provided with follow-up strategies to ensure patient success Users complete a feedback form on their experience and a battery of questionnaires to measure their progress

### Objectives

This paper aimed to provide a brief overview of the web-based system and to report on its usability from the perspectives of information and communications technology (ICT) experts and clinicians practicing in the field of hand therapy. Usability testing is a critical step in the development of online interventions and involves obtaining feedback to understand what is positive or negative about a system and identify existing gaps in content or functionality using iterative cycles of prototype alteration [58].

## Methods

### Design and Procedure

**Figure 1:**
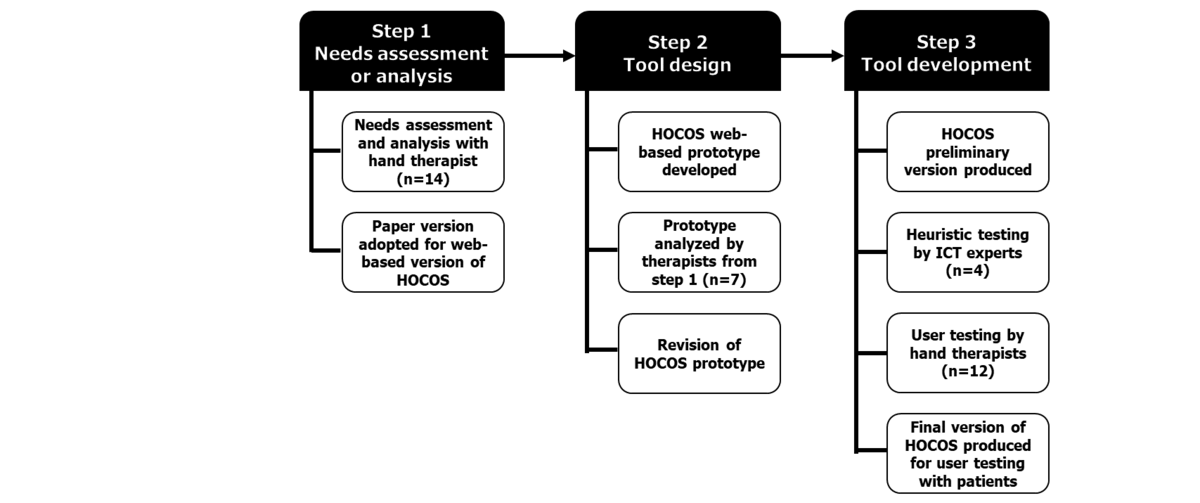
Diagram depicting the first 3 steps of Assessment or analysis, Design, Development, of the ADDIE Model. HOCOS: Hand Therapy Online Coping Skills; ICT: information and communications technology.

A mixed methods usability testing approach with semistructured interviews, observations, and questionnaires was undertaken, with iterative cycles to determine the usability of HOCOS and to further refine the prototype [59,60]. Participants were recruited using snowball sampling by asking key informants to suggest another participant who they believe is suitable for the study and introducing that person to the researcher [61]. This paper reported on step 3 of the ADDIE process (Figure 1). ADDIE is commonly used in instructional development as a systematic way to achieve the desired results [62].

### Participants

We recruited ICT experts online through the *Weebly support* (Weebly) portal to participate as heuristic evaluators in phase 1 of usability testing. In phase 2, PTs and OTs based in Ontario, Canada, and practicing in the clinical area of hand therapy were invited to participate in the study to enhance the development of HOCOS. Clinicians were messaged directly using contact details available to the public on the Canadian Society of Hand Therapy (CSHT) website. Interested participants contacted the research team directly by telephone or email and were provided with a letter of information and signed consent forms before data collection. Log-in access to the password-protected HOCOS website was provided free of charge.

### Procedure

One of the researchers, FB, facilitated data collection by conducting interviews, taking notes, and observing participants’ behavior. Appointments were made to meet with participants at the study site or at a desired destination within 2 hours of the study site. A brief description of the study was provided to each participant, with emphasis that the evaluation was about the content and functionality of the website. An explanation of cognitive interviews and information about privacy, and protection of the data collected were also provided. Before the interviews, demographic data, including age, gender, educational level, practice area, and use of technology, were collected. All participants were identified by pseudonyms to ensure anonymity [63]. According to Nielsen [56], 5 users are adequate to identify most usability problems. Current evidence shows that 80% of usability problems can be identified with 4 to 9 participants and 95% with 9 participants [64], thus we proposed a convenience sample of 12 participants for usability testing and to account for attrition. The usability testing protocol was approved by the Western University Health Sciences Research Ethics Board (no. 108064). Guided by steps 1 and 2 (Figure 1), we revised the prototype and developed a preliminary version of HOCOS that was tested by ICT experts (n=4) and therapists (n=12). No incentive was provided to participate in this study. Parking costs were covered for participants involved in face-to-face cognitive interviews.

#### Phase 1: Heuristic Testing

Heuristic testing is a usability inspection method completed by usability experts and involves evaluating an application to find usability problems, assigning them to a specific category of heuristics and ascribing a severity rating [58]. ICT experts were given a brief introduction to the background and rationale of the web portal under review and given instructions on how to conduct the heuristic testing. Between March and May 2018, the evaluators each separately conducted a heuristic evaluation of HOCOS through a page-by-page review of the website and noted *violations* where the interface did not conform to two sets of heuristics of predetermined criteria: the Monkman heuristics [65] and Health Literacy Online (HLO) checklist [66]. HLO was designed for the creation of usable online health content and comprises 35 separate criteria, categorized into 5 domains: write actionable content, display content clearly on the page, organize content and simplify navigation, engage users, and testing site with users with limited literacy skills [66]. The fifth domain was not factored in this study because this study focused on system design and development rather than implementation in practice. Monkman heuristics [65] comprises 11 checklist items and was designed for experienced heuristic evaluators by summarizing design guidelines from the HLO guide and incorporating research from electronic health (eHealth)/health literacy and usability literature [67]. The evaluators conferred using Skype clx and aggregated their results only after completing individual reviews. This phase resulted in the construction of a list of usability violations that were used to inform design changes before user testing commenced.

#### Phase 2: User Testing

User testing involved asking each participant to go through the website using the *thinking aloud* method [68], followed by a semistructured interview to elicit further feedback about user interaction. Each session was completed during a 1.5- to 2-hour face-to-face visit between September 2018 and March 2019. This enabled the researcher to capture the ongoing thought processes of the participants while going over the program and any difficulties encountered [69]. First, participants were required to log on to the website, read an introductory script, and familiarize themselves with the online learning environment using hyperlinks to move between pages. Next, the participants completed the following tasks: (1) logging in, (2) reading the introductory page, (3) completing a set of psychosocial outcome measures, (4) listening to an audio recording, (5) reading a PowerPoint presentation, (6) downloading a PDF or Word document script, (7) completing one activity in the workbook, (8) setting up an activity plan for homework, (9) finding contents by browsing, (10) finding contents by searching, (11) completing a feedback form, and (12) contacting the web manager. These tasks tested the user’s ability to follow the session plan and the amount of assistance required to use the online electronic tools. The facilitator did not offer any help during the tasks unless explicitly requested by the participants [68,70]. The facilitator encouraged the participants to talk about what they felt, saw, or thought while browsing through the website during the cognitive interviews. Verbal probes were also used to clarify the participant’s answers [70].

The facilitator also asked the participants to explain or demonstrate the information in the video related to the module that was reviewed, such as metal practice and breathing exercises in module 1 and module 5, respectively. The participant’s ability to follow the instructions correctly was observed, and any difficulties, doubts, and reports were documented using a 3-point scale (1=correctly demonstrated, 2=assistance required from an evaluator or replaying the video, and 3=difficulty demonstrating the activity correctly after being assisted) [56]. At the participant’s request, whole or specific areas of content were revisited. On the basis of the benchmark by Rubin and Chisnell [71], a task was classified as a usability problem requiring attention to remedy if more than 70% of participants were unable to complete the task. The system usability scale (SUS) questionnaire [72,73] was used to evaluate satisfaction. SUS comprises 10 open-ended, polarity balance–based questions with a 5-point Likert scale for responses. The average scores were categorized based on a descriptor rating scale [74]. Finally, the facilitator interviewed each participant using a semistructured interview guide (Multimedia Appendix 1) adopted from the study by Stinson et al [75] to obtain feedback about navigation, content, and layout at the end of the second cycle of user testing.

### Data Analysis

All interviews were audiotaped and transcribed verbatim in an anonymized format. The usability testing and interview data were analyzed together using triangulation [76]. Content analysis [77] of transcripts from the thinking aloud sessions, field notes, and feedback interviews was coded using predetermined codes related to usability issues (navigation, content, layout, learnability, errors, and satisfaction) after each iterative cycle. The interviews from the first cycle were analyzed and used to make minor modifications to the website before evaluation in the second cycle of testing. Very few modifications to the prototype were required after the second cycle of testing. To calculate the SUS score, the score contributions from each item are summed. Each item’s score contribution ranges from 0 to 4. For items 1, 3, 5, 7, and 9, the score contribution is the scale position minus 1. For items 2, 4, 6, 8, and 10, the contribution is 5 minus the scale position. Quantitative data from SUS (10 questions, each scored from 0 to 4 points) were transformed by multiplying by 2.5 to convert scores to a 0 to 100 range and categorized using adjective ratings [74]. The descriptive analysis (means and SD) of the quantitative data was conducted using Stata 13 software for Microsoft Office.

## Results

### Participants’ Characteristics

We enrolled four ICT experts to act as evaluators during heuristics evaluation, which meets the optimal requirement for detecting all usability problems [78]. During user testing, 26 clinicians agreed to participate in this study (14 for needs assessment, and 12 for usability testing). A total of 69% (18/26) of participants were female (Table 2). Most participants (17/26, 65%) had a background in occupational therapy, had at least 16 years of experience in hand therapy (10/26, 38%), and practiced in outpatient rehabilitation facility (10/26, 38%). Most therapists were very comfortable using a computer/tablet or internet. See Table 2 for participants’ characteristics.

**Table 2:** Demographic and computer and internet use characteristics of therapists participating in needs assessment/analysis and usability testing of the study (N=26).

Demographics			Needs assessment (n=14)	Usability testing (n=12)
**Age (years), n (%)**				
	21-30		3 (21)	2 (17)
	30-40		5 (35)	2 (17)
	40-50		2 (14)	3 (25)
	>50		4 (29)	5 (42)
**Gender, n (%)**				
	Male		6 (43)	2 (17)
	Female		8 (57)	10 (83)
	Prefer not to say		0 (0)	0 (0)
**Profession, n (%)**				
	Occupational therapists		10 (71)	7 (58)
	Physiotherapists		4 (29)	5 (42)
**Education, n (%)**				
	Entry level (baccalaureate degree)		4 (29)	3 (25)
	Entry level (master degree)		8 (57)	8 (57)
	PhD		2 (15)	1 (8)
**Work experience (years), n (%)**				
	<5		2 (15)	0 (0)
	10-15		4 (29)	3 (25)
	16-20		6 (43)	4 (33)
	>20		2 (15)	5 (42)
**Practice setting, n (%)**				
	Private practice		2 (15)	2 (17)
	Acute care		3 (21)	3 (25)
	Inpatient rehabilitation		2 (15)	3 (25)
	Outpatient rehabilitation		6 (42.8)	4 (33)
	Other (teaching)		1 (6)	0 (0)
**Employment, n (%)**				
	Full time		9 (64)	9 (75)
	Part time		3 (21)	3 (25)
	Casual		1 (6)	0 (0)
**Information about computer use, n (%)**				
	**Computer/tablet use at home**			
		Yes	12 (85)	12 (100)
		No	2 (15)	0 (0)
	**Computer/tablet use at work**			
		Yes	14 (100)	12 (100)
		No	0 (0)	0 (0)
	**Hours spent on computer/tablet each week**			
		≤5	0 (0)	0 (0)
		>5	14 (100)	12 (100)
	**Hours spent on the internet each week**			
		≤5	5 (35)	4 (33)
		>5	9 (65)	8 (67)
	**Comfort level on computer/tablet**			
		Not at all comfortable	0 (0)	0 (0)
		A little comfortable	0 (0)	0 (0)
		Comfortable	4 (29)	4 (33)
		Very comfortable	10 (71)	8 (67)
	**Comfort level on the internet**			
		Not at all comfortable	0 (0)	0 (0)
		A little comfortable	2 (14)	0 (0)
		Comfortable	4 (29)	4 (33)
		Very comfortable	8 (57)	8 (67)

### Phase 1: Heuristic Testing

The heuristic evaluation of HOCOS against the HLO checklist identified violations in 15 of the 35 criteria with violations seen across all domains (Multimedia Appendix 2). Domain 1 showed violations in 2 of the 7 criteria. There were 4 violations in the 13 criteria for domain 2. Most of the violations were represented in domain 3, with 6 of the 10 violations reported. Violations included (1) the home page image not representing the context of the website, (2) lack of a search function, and (3) links that are difficult to differentiate from the surrounding text or other graphic elements. Corrections were made and included adding a welcome image on the home page, adding a search function, and creating a box around link icons. Domain 4 revealed 3 violations in the 5 criteria because of heavy reliance on text-based information, lack of quizzes or forms, and lack of social media sharing options. We included more pictures and reduced the words per page, creating a separate link for the form. We decided against adding a social media link because of privacy concerns and the sensitive nature of psychosocial issues. Evaluation of HOCOS using Monkman heuristics identified violations in 5 of the 11 criteria (Multimedia Appendix 3) including lack of options for tailoring information to the user, poor use of plain language including medical jargon and Gunning Fog readability index greater than 8 [79,80], information in multiple languages, few succinct summaries versus more detailed information, need for scrolling to find important information, and poor communication of risks. The remaining violations were managed by adding activities that could be personalized, editing the content for therapists and patients using the Gunning Fog index, creating a summary of key points in the slides, adding an icon to important information, and adding a disclaimer to express inherent risks and benefits of the program.

### Phase 2: User Testing

This included findings from the user task performance and cognitive interviews (thinking aloud) components of usability testing of the HOCOS.

#### Task Performance

We measured user performance based on ease of navigating through the site, assessing the ease of learning for a first-time user without familiarity with the interface, and the frequency and importance of errors. Errors observed during usability testing were reported in 3 categories: completed with ease, completed with help, and not completed [75]. The performance of the 10 tasks is presented in Table 3. In summary, seven tasks were completed easily by participants: logging in, browsing, reading the introductory pages, listening to audio files, reading PowerPoint presentations, filling a homework plan, contacting the researchers, and downloading a document. The remaining five tasks revealed difficulties with usability. Navigation errors were defined as failures to locate functions, excessive keystrokes, or failures to follow recommended screen flow [81]. Five participants were not able to find the *assessment* page to fill outcome measures. The page was accessible through the *resources* page, although the opening comments on the page highlighted contents on the resource page. There were 6 participants who did not realize that the workbook contained both educational information and homework despite text alongside the introduction highlighting different module assignments.

Control usage errors were defined as improper toolbars or entry field usage [75]. Five participants were unable to identify the icons for submitting answers to some activities on the modules. This error was corrected by typing *click on the link to write your answers* on the link to provide answers. Providing feedback using the website form was the most difficult task for participants. Users did not click on the next page at the end of every module where the feedback form was placed. We included a text highlighting where to find the feedback form on the module’s introductory page and on the final page of every module. Presentation errors were defined as failures to locate and properly act upon desired information or selection errors because of labeling obscurities [75].

**Table 3:** Task performance findings during usability testing (N=12).

Task performance		Completed, n (%)		Not completed, n (%)	
		With ease	With help		
**Cycle 1**					
	Log in to the website	8 (66)	3 (25)		1 (8)
	Read information on the home page and each module’s introductory page	10 (83)	2 (17)		N/A^a^
	Complete a questionnaire from the list of outcome measures	4 (33)	3 (25)		5 (42)
	Listen to an audio recording	8 (66)	4 (33)		N/A
	Read a PowerPoint slide	8 (66)	3 (25)		1 (8)
	Download a PDF or Word document of a workbook or PowerPoint slide	10 (83)	2 (17)		N/A
	Complete 1 activity in a workbook	4 (33)	2 (17)		6 (50)
	Set up an activity plan for homework	7 (58)	3 (25)		2 (17)
	Find content of interest by browsing	9 (42)	2 (25)		1 (8)
	Find content of interest by searching	4 (33)	3 (25)		5 (42)
	Complete a feedback form	2 (17)	3 (25)		7 (58)
	Contact the website manager	8 (33)	4 (50)		N/A
**Cycle 2**					
	Log in to the website	12 (100)	N/A		N/A
	Read information on the home page and each module’s introductory page	12 (100)	N/A		N/A
	Complete a questionnaire from the list of outcome measures	10 (83)	2 (17)		N/A
	Listen to an audio recording	8 (66)	4(33)		N/A
	Read a PowerPoint slide	12 (100)	N/A		N/A
	Download a PDF or Word document of a workbook or PowerPoint slide	12 (100)	N/A		N/A
	Complete 1 activity in a workbook	8 (66)	4 (33)		N/A
	Set up an activity plan for homework	10 (83)	2 (17)		N/A
	Find content of interest by browsing	9 (75)	3 (25)		N/A
	Find content of interest by searching	8 (66)	4 (33)		N/A
	Complete a feedback form	9 (75)	3 (25)		N/A
	Contact the website manager	12 (100)	N/A		N/A

^a^N/A: not applicable.

Searching was a bit of a challenge for 5 participants because they did not know what to search for, unsure of search terms to use, or struggled to come up with a health topic in the context of the website. Participants completed the 12 tasks in phase 2 at the end of the second cycle of testing.

#### Cognitive Interviews

The key usability findings from the thinking aloud interviews were organized into the following themes: design aesthetics, content, functionality and features, and desire for future use. Multimedia Appendix 4 shows participants’ quotes from cognitive interviews.

#### Design Esthetics

Overall design aesthetics was critical to enhancing engagement and motivation to use the website and related to the layout, navigation, visual assets, and appeal. Participants liked the idea of different textures, colors, and cultures represented in the graphics. It was suggested that the font sizes should be set at size 14 to 16, and a large amount of information should be grouped and broken up with visual assets (graphics and illustrations). In response, we divided the PowerPoint slides into parts A and B and/or C to reduce information overload and reduce the feeling of being overwhelmed. Part C was created as an addendum with the caption, *Please see part C for a deeper learning on this topic.* Users also recommended that the most important message on each page should be at the top of the page. As the modules were stand-alone content, the participants suggested that a decision tree or matrix would reduce the burden of prescribing the appropriate module to patients based on their presentation and treatment goals. In response, we created a matrix with information on key learning points, indications, and contraindications for each module. For example, patients with paradoxical responses to visualization avoid thinking about their hand injury, and those focused on the loss may find mental imagery distressing. Changes were also suggested to some features to increase user interest and reduce negative responses. For example, we changed the titles *Mental Practice* to *Picturing My Movement*, *Thought Reprocessing* to *Healthy Thinking*, and *Board of Directors* to *Thinking Traps*.

#### Content

Program content was described in terms of completeness, understandability, quality, credibility, relevance, and interest. The comments on program content, such as texts, images, and multimedia components, were generally positive. The layout structure of presenting information in different formats and having a summary of key points after each lesson was valued. All participants judged that the site content was relevant and credible. Generally, participants were pleased with the completeness of the website, but additional content was suggested. Examples of additions included creating reflective pieces to improve engagement with the slideshows and linking activities under *Mental Practice* to portray the multisensory nature of hand movement. HOCOS was created with a focus on understandability, and all text developed to meet grade 6 to 7 reading levels. Most participants valued this consideration and commented that the information, language level, and medical term explanations were helpful in furthering understanding of topics that were unclear or new to them. However, some of the language used still had to be changed to conjure everyday talk and meet societal norms such as changing wife/husband to spousal partner and routine to day-to-day. Several language changes were made to clarify meaning, such as changing tissues to body, thought record to thought journal, healthy to uninjured, and food for thought to pause-stop-think.

#### Functionality and Features

These refer to the adaptive and interactive features on the website and included module 1 to 5 audio clips, printable PDF information forms for patient and clinician users, and videos of simulated patients completing module activities. It was agreed that these features allowed for an increased level of personalization of HOCOS content to meet the individual needs of the users. To further enhance participants’ motivation and engagement, we added the following functions: interactive questions (quizzes after each PowerPoint presentation), an *Ask an Expert* link to allow users to send an email question to the web developers and a goal plan journal to keep track of goals and activities. Participants suggested having features that allowed the program to support social interactions among participants, such as a discussion board. However, because of budgetary and time constraints, we were unable to include these functions in HOCOS. Other features that were introduced to help patients incorporate the new information to their daily routine was the *How to Make It Work Guide*.

#### Desire for Future Use

Overall, participants received HOCOS very well and expressed a desire to use the program in the future. It was agreed that the website would be especially useful if available to patients from initial contact for presurgical screening with surgeons or immediately after surgery in acute care. The therapists commented that they valued the fact that the site content focused on supplementing current hand therapy practice for patients struggling with psychosocial issues. The accompanying navigation of the workflow would make it easy to prioritize programs for their patients. Most participants suggested that collaboration with the CSHT and hand programs in Ontario would help facilitate increased uptake in the hand therapy community.

#### System Usability Scale and User Satisfaction

The SUS scores from both cycles of usability testing are listed in Table 4. Scores above 68 (SD 12.5) indicate above-average usability [82]. The mean SUS score for this study improved from 62.5 (SD 8.5) to 84 (SD 8.2), indicating that the average participants were highly satisfied with the usability of this online learning tool on all items of the SUS questionnaire, in terms of learnability, efficiency, memorability, errors, and satisfaction [56]. After addressing cycle 2 usability issues, we made some revisions to the final version of HOCOS. Specifically, a do-it-yourself (DIY) guide was created to support each module, a *Go to homepage* tab was created as a signpost to the respective sessions after logging in, and a navigation tutorial video and informational videos on the clinical impact of psychosocial factors on hand and upper limb injuries were created. Finally, we included patient-friendly resources on chronic pain, problem solving, time management, and a sleep guide. Overall, therapists found that HOCOS was a detailed and helpful learning resource for therapists and patients. Participants liked the web layout, tabs for modules, and resource page. There were no reported harms or unintended effects on participants, privacy breaches, or technical problems during usability testing. Overall, HOCOS system usability improved from good to excellent based on adjective rating scale described by Bangor et al [74].

**Table 4:** System usability scale (N=12).

Questionnaire items	Cycle 1, mean (SD)^a^	Cycle 2, mean (SD)^a^
1. I think that I would like to use this website frequently (+)^b^	3 (0.8)	4 (0.5)
2. I found the website unnecessarily complex (−)^c^	2 (0.7)	3 (0.9)
3. I thought the website was easy to use (+)^b^	2 (0.6)	3 (0.0)
4. I think that I would need the support of a technical person to be able to use this website (−)^c^	3 (1.08)	4 (0.5)
5. I found the various functions in the product were well integrated (+)^b^	3 (0.5)	3 (0.5)
6. I thought there was too much inconsistency in this website (−)^c^	3 (0.4)	4 (0.5)
7. I imagine that most people would learn to use this product very quickly (+)^b^	2 (0.7)	3 (0.4)
8. I found the website very awkward to use (−)^c^	3 (0.5)	4 (0.5)
9. I felt very confident using the website (+)^b^	2 (0.4)	3 (0.4)
10. I needed to learn a lot of things before I could get going with this website (−)^c^	2 (0.9)	3 (0.4)
Total score of items 1, 3, 5, 7, and 9	2.4 (0.5)	3.2 (0.6)
Total score of items 2, 4, 6, 8, and 10	2.6 (0.7)	3.6 (0.5)
Total score	25 (3.4)	34 (3.2)
SUS^d^ score^e^	62 (8.5)	84 (8.2)

^a^Rating scale, 1=strongly disagree and 5=strongly agree.

^b^For items 1, 3, 5, 7, and 9, the score contribution is the scale position minus 1.

^c^For items 2, 4, 6, 8, and 10, the contribution is 5 minus the scale position.

^d^SUS: system usability scale.

^e^SUS score=total score×2.5.

#### Final Version of Hand Therapy Online Coping Skills

**Figure 2:**
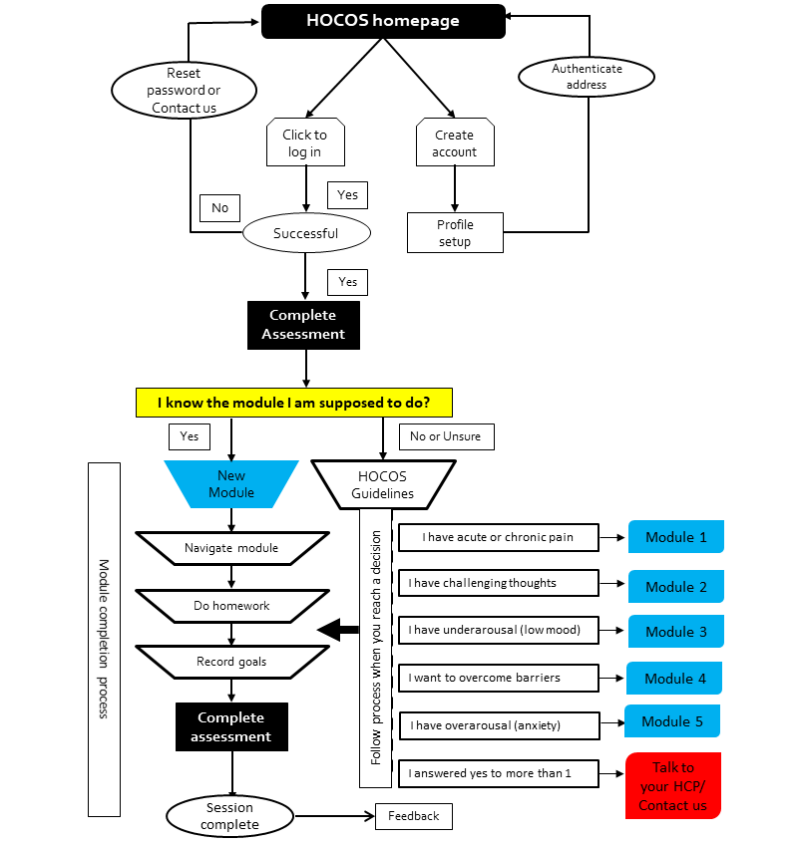
Navigation workflow of the Hand Therapy Online Coping Skills (HOCOS) training program. HCP: health care professional.

**Figure 3:**
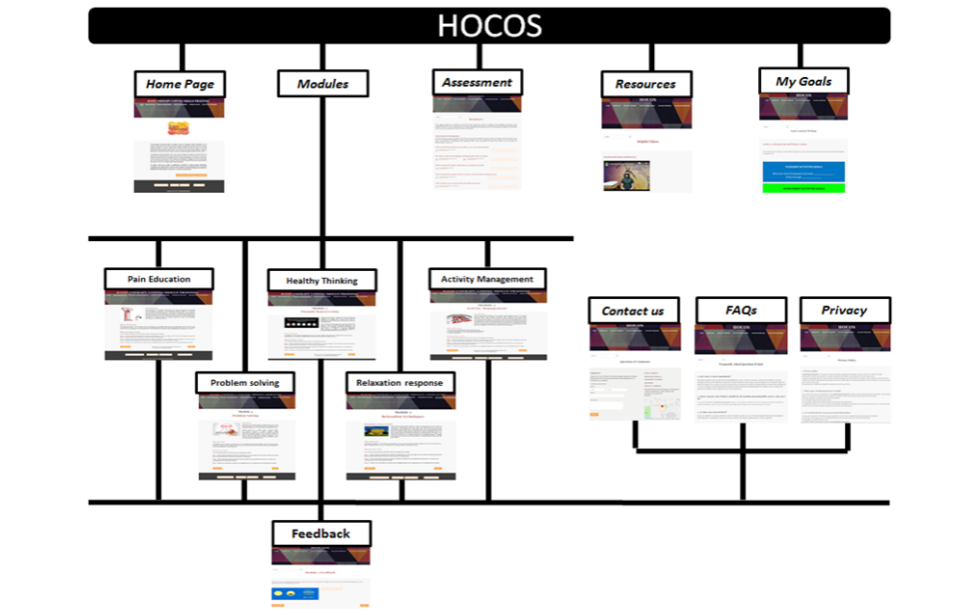
Screenshot of webpages showing the architecture of the website. FAQs: frequently answered questions; HOCOS: Hand Therapy Online Coping Skills.

The final version of HOCOS was built on the Weebly platform, customized and styled using platform add-ons and publicly available pictures on Creative Commons. The platform included a landing page, a resource library, tabs for each of the modules, a feedback page, an assessment page, a goals page, and therapist- or patient-specific resources (Figure 2). Multimedia Appendix 1 gives a brief overview of the HOCOS content. Sessions can be accessed by logging in and completed using a suggested timetable. Figure 3 shows a navigation pathway to complete the 5 modules. Each module can be completed as stand-alone materials based on patient presentation. However, we recommend that every patient complete the introductory and pain education sections. Completing all five modules is projected to take approximately 6 to 8 weeks based on the structure of similar coping skill programs [83]. HOCOS is designed to be beneficial for both acute and chronic hand injuries. A therapist manual was also developed based on participant feedback.

## Discussion

### Principal Findings

The overall objectives of this paper were to provide an overview of HOCOS and report the findings from usability testing with ICT experts and clinicians practicing hand therapy. HOCOS is designed to help patients with hand and upper limb injuries learn how to better manage psychosocial issues. The uniqueness of HOCOS is an interface design that offers learning opportunities to both clinicians and patients. Overall, therapists were pleased with the objective and content of HOCOS and found it a useful resource for meeting patients’ needs in hand therapy.

### Usability Testing

Formal usability testing is a key process required to ensure the relevance of content and make the website easy to use, learn, efficient, and acceptable to users [75]. Usability testing uncovered several violations during heuristic testing with ICT experts. Furthermore, user performance errors and areas for enhancing user satisfaction were also identified by therapists during user testing. Therapists reported some positive features of the website including being simple, user-friendly, and engaging and having a functional design that was accessible on several browsers following usability testing. Several changes were made to the online portal that corrected the errors uncovered and improved overall user satisfaction. Although HOCOS was initially designed for all modules to be completed together, feedback from clinicians highlighted the benefits of having modules as stand-alone options to reduce potential participant burden. Hand therapists expressed confidence that patients could execute the activities in the workbook, especially with the DIY guide. Testing also demonstrated that the primary condition of the patients determined the modules that therapists chose to introduce and apply in clinical practice. This process was enhanced by providing a guide on how to use the features, when to introduce the modules, and how the website may fit within the broader tool kits used in hand therapy.

This study contributes to the dearth of literature on the usability testing of web-based portals developed for managing psychosocial factors in orthopedic hand and upper extremity services. Chad-Friedman et al [84] reported the use of an online interface designed to deliver a brief 60-second mindfulness exercise for hand and upper limb pain with improvements in state anxiety, pain intensity, distress, anxiety, depression, and anger after watching the video. In another study by Westernberg et al [42], a free online mindfulness-based video exercise was targeted at individuals with upper extremity conditions and psychosocial problems. Study findings reported improvement in momentary pain, anxiety, depression, and anger in patients with low levels of pain and psychologic distress. Similarly, Vranceanu et al [85] described the Toolkit for Optimal Recovery (TOR), a 4-session, live video, and manualized program informed by the fear-avoidance model to prevent chronic pain in at-risk adults with orthopedic injuries. TOR combines relaxation response with CBT, Acceptance and Commitment therapy skills. HOCOS provides a larger platform offering multiple options to therapists and patients using concepts from pain education science, relaxation response and behavior change techniques. On the basis of postcognitive interview feedback, therapists involved in this study preferred an online program that teaches patients how to change maladaptive cognitions and not simply accept such thoughts for long term effects. To close existing gaps in the literature, HOCOS was designed using CBT principles which teaches patients how to challenge automatic thoughts by holding them up to disproving evidence and then change them into different thoughts [86,87]. CBT begins by identifying a primary treatment goal and continuous striving to meet those goals [88]. HOCOS also provides modules that can be targeted at psychosocial problems associated with acute to subacute and chronic hand and upper limb conditions with therapist guidance. This is important because the untargeted use of psychological interventions in hand therapy and when self-directed by patients has been shown to demonstrate no benefits [89].

Dissemination of evidence-based therapies remains poor in routine practice [90]. Although allied health care professionals (HCPs) are aware of the benefits of incorporating psychological interventions within their practice, they feel insufficiently trained to optimize their use of such interventions [91]. Barriers to practicing the evidence-based therapies include a lack of access to resources that contain such evidence [92,93] and limited usable formats of the evidence [94]. Training hand therapists to manage the physical and psychological sequelae of hand and upper limb conditions using HOCOS would increase their knowledge of psychosocial interventions and build their capacity and confidence to deliver it in clinical practice.

### Limitations

Our study should be viewed with consideration of certain limitations encountered. The study was conducted among hand therapists in Ontario, and most participants were comfortable using the computer and the internet, which limit the generalizability of the study results. This may not be representative of the end users, such as patients seen in most hand therapy clinics. In recruiting participants for this study, we chose snowball sampling, a form of convenience sampling. This increases the risk of compiling a nonrepresentative sample. We planned to create an online platform that is user friendly for a significant portion of patients with hand and upper limb injuries who are mostly elderly [95], low skilled [96], and with less education [97]. These groups of individuals tend to be less computer literate, and to this end, we did our best to incorporate recommendations to ensure accessibility and ease of use in the web design and simplify the user experience [66]. This included a larger font size, white space around texts, and a simple color scheme to enhance readability.

The presence of one of the researchers (FB) during the usability testing sessions may have affected the behavior of end users conducting the testing. The participants may have felt reluctant to be critical despite encouragement to highlight both weak and strong features of the website. Furthermore, we were unable to test the HOCOS website in the context of the patient-user’s experience to gain a comprehensive view of the system’s functioning in a clinical setting because of financial and time constraints. This needs to be addressed in future research by examining the effectiveness of HOCOS in a randomized controlled trial to determine if the present system design can contribute to improved outcomes in practice.

User testing of an online intervention should include the ultimate end users, including patients, to allow for the examination of factors related to participants (age, gender, and education), disease (severity and duration of symptoms), and experience (access to and comfort with using the internet and computers) [75,98]. On the basis of ergonomic quality and safety principles, it has been recommended that prototypes of eHealth interventions should be fully inspected and walked through by HCPs before exposure to potentially vulnerable user groups such as individuals with significant psychosocial problems after a hand injury [99]. Financial and time constraints were significant barriers to testing HOCOS in patients with hand injuries. The next phase of the project is to evaluate the impact of HOCOS training on the actual implementation of the program on patients. We plan to carry out further testing in a proof-of-concept study to establish if individuals with hand and upper limb conditions and psychosocial problems are willing and able to complete the HOCOS program, complete the activities correctly, and adhere to the program principles.

### Conclusions

This study provides initial support for the usability of HOCOS. Ensuring that therapists were involved in the design and development process of HOCOS enhanced the user-centeredness and user-friendliness of the website. Usability testing during the formative stage of eHealth intervention development is necessary to ensure that online interventions are effective and acceptable to potential users. HOCOS has the potential to increase access and acceptability of coping skills training programs for many individuals with hand and upper limb injuries who are not able to receive hospital- or clinic-based treatment psychotherapy. We plan to conduct a pilot study to determine the feasibility of the website for adults with hand and upper limb injuries and further refine the tool for a fully powered randomized controlled trial. If effective in improving outcomes, this program could be used as a template to develop more interventions targeting the psychosocial challenges confronting individuals with hand and upper limb injuries.

## Appendix

Multimedia Appendix 1 Semistructured interview guide for conducting cognitive interviews during usability testing.

Multimedia Appendix 2 Heuristic evaluation of Hand Therapy Online Coping Skills by information and communications technology experts using general and readability guidelines of the Health Literacy Online checklist.

Multimedia Appendix 3 Heuristic evaluation of Hand Therapy Online Coping Skills by information and communications technology experts using health-specific usability guidelines based on Monkman heuristics.

Multimedia Appendix 4 Sample comments for each of the themes derived from analysis of the cognitive interview and feedback interview transcripts.

## Abbreviations

ADDIE: Analysis, Design, Development, Implementation, and Evaluation
CBT: cognitive behavioral therapy
CIHR: Canadian Institutes Health Research
CSHT: Canadian Society of Hand Therapy
DIY: do-it-yourself
eHealth: electronic health
HCP: health care professional
HLO: Health Literacy Online
HOCOS: Hand Therapy Online Coping Skills
ICT: information and communications technology
MSK: musculoskeletal
OT: occupational therapist
PT: physiotherapist
SUS: system usability scale
TOR: Toolkit for Optimal Recovery

## References

[1] Junqueira GD, Lima AL, Boni R, Almeida JC, Ribeiro RS, Figueiredo LA (2017). Incidence of acute trauma on hand and wrist: a retrospective study. Acta Ortop Bras.

[2] Turkington C, Dempster M, Maguire J (2018). Adjustment to hand injury: cross-sectional survey exploring adjustment in relation to illness perceptions and coping strategies. J Hand Ther.

[3] de Putter CE, Selles RW, Polinder S, Panneman MJ, Hovius SE, van Beeck EF (2012). Economic impact of hand and wrist injuries: health-care costs and productivity costs in a population-based study. J Bone Joint Surg Am.

[4] de Jong JP, Nguyen JT, Sonnema AJ, Nguyen EC, Amadio PC, Moran SL (2014). The incidence of acute traumatic tendon injuries in the hand and wrist: a 10-year population-based study. Clin Orthop Surg.

[5] Vranceanu A, Bachoura A, Weening A, Vrahas M, Smith RM, Ring D (2014). Psychological factors predict disability and pain intensity after skeletal trauma. J Bone Joint Surg Am.

[6] Vranceanu A, Jupiter JB, Mudgal CS, Ring D (2010). Predictors of pain intensity and disability after minor hand surgery. J Hand Surg Am.

[7] Rumsey N, Harcourt D (2004). Body image and disfigurement: issues and interventions. Body Image.

[8] Hannah SD (2011). Psychosocial issues after a traumatic hand injury: facilitating adjustment. J Hand Ther.

[9] Bot AG, Bossen JK, Mudgal CS, Jupiter JB, Ring D (2014). Determinants of disability after fingertip injuries. Psychosomatics.

[10] Ring D (2011). The role of science and psychology in optimizing care of hand illness. J Hand Ther.

[11] Chan J, Spencer J (2004). Adaptation to hand injury: an evolving experience. Am J Occup Ther.

[12] Keir PJ, Zuniga AF, Mulla DM, Somasundram KG (2019). Relationships and mechanisms between occupational risk factors and distal upper extremity disorders. Hum Factors.

[13] Shi Q, Sinden K, MacDermid JC, Walton D, Grewal R (2014). A systematic review of prognostic factors for return to work following work-related traumatic hand injury. J Hand Ther.

[14] Roesler ML, Glendon AI, O'Callaghan FV (2013). Recovering from traumatic occupational hand injury following surgery: a biopsychosocial perspective. J Occup Rehabil.

[15] Daud AZ, Yau MK, Barnett F, Judd J, Jones RE, Nawawi RF (2016). Integration of occupation based intervention in hand injury rehabilitation: a randomized controlled trial. J Hand Ther.

[16] Bobos P, Nazari G, Szekeres M, Lalone EA, Ferreira L, MacDermid JC (2019). The effectiveness of joint-protection programs on pain, hand function, and grip strength levels in patients with hand arthritis: a systematic review and meta-analysis. J Hand Ther.

[17] Menta R, Randhawa K, Côté P, Wong JJ, Yu H, Sutton D, Varatharajan S, Southerst D, D'Angelo K, Cox J, Brown C, Dion S, Mior S, Stupar M, Shearer HM, Lindsay GM, Jacobs C, Taylor-Vaisey A (2015). The effectiveness of exercise for the management of musculoskeletal disorders and injuries of the elbow, forearm, wrist, and hand: a systematic review by the Ontario protocol for traffic injury management (OPTIMa) collaboration. J Manipulative Physiol Ther.

[18] Sultana SS, MacDermid JC, Grewal R, Rath S (2013). The effectiveness of early mobilization after tendon transfers in the hand: a systematic review. J Hand Ther.

[19] Szekeres M, MacDermid JC, Birmingham T, Grewal R, Lalone E (2017). The effect of therapeutic whirlpool and hot packs on hand volume during rehabilitation after distal radius fracture: a blinded randomized controlled trial. Hand (N Y).

[20] Bennell KL, Ahamed Y, Jull G, Bryant C, Hunt MA, Forbes AB, Kasza J, Akram M, Metcalf B, Harris A, Egerton T, Kenardy JA, Nicholas MK, Keefe FJ (2016). Physical therapist-delivered pain coping skills training and exercise for knee osteoarthritis: randomized controlled trial. Arthritis Care Res (Hoboken).

[21] Das DS, Vranceanu A, Ring DC (2013). Contribution of kinesophobia and catastrophic thinking to upper-extremity-specific disability. J Bone Joint Surg Am.

[22] Farzad M, Asgari A, Dashab F, Layeghi F, Karimlou M, Hosseini SA, Rassafiani M (2015). Does disability correlate with impairment after hand injury?. Clin Orthop Relat Res.

[23] Roh YH, Lee BK, Noh JH, Oh JH, Gong HS, Baek GH (2014). Effect of anxiety and catastrophic pain ideation on early recovery after surgery for distal radius fractures. J Hand Surg Am.

[24] Roh YH, Noh JH, Oh JH, Gong HS, Baek GH (2015). To what degree do pain-coping strategies affect joint stiffness and functional outcomes in patients with hand fractures?. Clin Orthop Relat Res.

[25] Briet JP, Hageman MG, Overbeek CL, Mudgal C, Ring DC, Vranceanu A (2016). Factors associated with met expectations in patients with hand and upper extremity disorders: a pilot study. Psychosomatics.

[26] Groot D, van Hooff ML, Kroeze RJ, Monshouwer M, O'Dowd J, Horsting P, Spruit M (2019). Long-term results of an intensive cognitive behavioral pain management program for patients with chronic low back pain: a concise report of an extended cohort with a minimum of 5-year follow-up. Eur Spine J.

[27] Richmond H, Hall AM, Copsey B, Hansen Z, Williamson E, Hoxey-Thomas N, Cooper Z, Lamb SE (2015). The effectiveness of cognitive behavioural treatment for non-specific low back pain: a systematic review and meta-analysis. PLoS One.

[28] Tong F, Dannaway J, Enke O, Eslick G (2020). Effect of preoperative psychological interventions on elective orthopaedic surgery outcomes: a systematic review and meta-analysis. ANZ J Surg.

[29] Ismail A, Moore C, Alshishani N, Yaseen K, Alshehri MA (2017). Cognitive behavioural therapy and pain coping skills training for osteoarthritis knee pain management: a systematic review. J Phys Ther Sci.

[30] Anheyer D, Haller H, Barth J, Lauche R, Dobos G, Cramer H (2017). Mindfulness-based stress reduction for treating low back pain: a systematic review and meta-analysis. Ann Intern Med.

[31] Mariano TY, Urman RD, Hutchison CA, Jamison RN, Edwards RR (2018). Cognitive behavioral therapy (CBT) for subacute low back pain: a systematic review. Curr Pain Headache Rep.

[32] Lauche R, Cramer H, Dobos G, Langhorst J, Schmidt S (2013). A systematic review and meta-analysis of mindfulness-based stress reduction for the fibromyalgia syndrome. J Psychosom Res.

[33] Rolving N, Sogaard R, Nielsen CV, Christensen FB, Bünger C, Oestergaard LG (2016). Preoperative cognitive-behavioral patient education versus standard care for lumbar spinal fusion patients: economic evaluation alongside a randomized controlled trial. Spine (Phila Pa 1976).

[34] das Nair R, Anderson P, Clarke S, Leighton P, Lincoln NB, Mhizha-Murira JR, Scammell BE, Walsh DA (2016). Home-administered pre-surgical psychological intervention for knee osteoarthritis (HAPPiKNEES): study protocol for a randomised controlled trial. Trials.

[35] Smittenaar P, Erhart-Hledik JC, Kinsella R, Hunter S, Mecklenburg G, Perez D (2017). Translating comprehensive conservative care for chronic knee pain into a digital care pathway: 12-week and 6-month outcomes for the hinge health program. JMIR Rehabil Assist Technol.

[36] Room J, Hannink E, Dawes H, Barker K (2017). What interventions are used to improve exercise adherence in older people and what behavioural techniques are they based on? A systematic review. BMJ Open.

[37] Brunner E, de Herdt A, Minguet P, Baldew S, Probst M (2013). Can cognitive behavioural therapy based strategies be integrated into physiotherapy for the prevention of chronic low back pain? A systematic review. Disabil Rehabil.

[38] Hall A, Richmond H, Copsey B, Hansen Z, Williamson E, Jones G, Fordham B, Cooper Z, Lamb S (2018). Physiotherapist-delivered cognitive-behavioural interventions are effective for low back pain, but can they be replicated in clinical practice? A systematic review. Disabil Rehabil.

[39] Bot AG, Bekkers S, Arnstein PM, Smith RM, Ring D (2014). Opioid use after fracture surgery correlates with pain intensity and satisfaction with pain relief. Clin Orthop Relat Res.

[40] Vranceanu A, Riklin E, Merker VL, Macklin EA, Park ER, Plotkin SR (2016). Mind-body therapy via videoconferencing in patients with neurofibromatosis: an RCT. Neurology.

[41] Beleckas CM, Wright M, Prather H, Chamberlain A, Guattery J, Calfee RP (2018). Relative prevalence of anxiety and depression in patients with upper extremity conditions. J Hand Surg Am.

[42] Westenberg RF, Zale EL, Heinhuis TJ, Özkan S, Nazzal A, Lee S, Chen NC, Vranceanu A (2018). Does a brief mindfulness exercise improve outcomes in upper extremity patients? A randomized controlled trial. Clin Orthop Relat Res.

[43] Kumar V, Sattar Y, Bseiso A, Khan S, Rutkofsky IH (2017). The effectiveness of internet-based cognitive behavioral therapy in treatment of psychiatric disorders. Cureus.

[44] Garg S, Garg D, Turin TC, Chowdhury MF (2016). Web-based interventions for chronic back pain: a systematic review. J Med Internet Res.

[45] Terpstra JA, van der Vaart R, van Koulil SS, van Dam A, Rosmalen JG, Knoop H, van Middendorp H, Evers AW (2018). Becoming an eCoach: training therapists in online cognitive-behavioral therapy for chronic pain. Patient Educ Couns.

[46] Cowell I, O'Sullivan P, O'Sullivan K, Poyton R, McGregor A, Murtagh G (2019). The perspectives of physiotherapists on managing nonspecific low back pain following a training programme in cognitive functional therapy: a qualitative study. Musculoskeletal Care.

[47] Stiles-Shields C, Kwasny MJ, Cai X, Mohr DC (2014). Therapeutic alliance in face-to-face and telephone-administered cognitive behavioral therapy. J Consult Clin Psychol.

[48] Pihlaja S, Stenberg J, Joutsenniemi K, Mehik H, Ritola V, Joffe G (2018). Therapeutic alliance in guided internet therapy programs for depression and anxiety disorders-a systematic review. Internet Interv.

[49] Haug T, Nordgreen T, Öst LG, Tangen T, Kvale G, Hovland OJ, Heiervang ER, Havik OE (2016). Working alliance and competence as predictors of outcome in cognitive behavioral therapy for social anxiety and panic disorder in adults. Behav Res Ther.

[50] Cooper Z, Bailey-Straebler S, Morgan KE, O'Connor ME, Caddy C, Hamadi L, Fairburn CG (2017). Using the internet to train therapists: randomized comparison of two scalable methods. J Med Internet Res.

[51] Everitt HA, Landau S, O'Reilly G, Sibelli A, Hughes S, Windgassen S, Holland R, Little P, McCrone P, Bishop F, Goldsmith K, Coleman N, Logan R, Chalder T, Moss-Morris R, ACTIB Trial Group (2019). Assessing telephone-delivered cognitive-behavioural therapy (CBT) and web-delivered CBT versus treatment as usual in irritable bowel syndrome (ACTIB): a multicentre randomised trial. Gut.

[52] Hageman MG, Anderson J, Blok R, Bossen JK, Ring D (2015). Internet self-diagnosis in hand surgery. Hand (N Y).

[53] Kelly JD (2018). CORR insights: does a brief mindfulness exercise improve outcomes in upper extremity patients? a randomized controlled trial. Clin Orthop Relat Res.

[54] Holden RJ, Karsh B (2010). The technology acceptance model: its past and its future in health care. J Biomed Inform.

[55] Levac D, Glegg SM, Camden C, Rivard LM, Missiuna C (2015). Best practice recommendations for the development, implementation, and evaluation of online knowledge translation resources in rehabilitation. Phys Ther.

[56] Srikesavan CS, Williamson E, Eldridge L, Heine P, Adams J, Cranston T, Lamb SE (2017). A web-based training resource for therapists to deliver an evidence-based exercise program for rheumatoid arthritis of the hand (iSARAH): design, development, and usability testing. J Med Internet Res.

[57] Bennell KL, Egerton T, Pua Y, Abbott JH, Sims K, Buchbinder R (2011). Building the rationale and structure for a complex physical therapy intervention within the context of a clinical trial: a multimodal individualized treatment for patients with hip osteoarthritis. Phys Ther.

[58] Nielsen J, Nielsen J, Mack RL (1994). Heuristic evaluation. Usability Inspection Methods.

[59] Allen M, Currie LM, Bakken S, Patel VL, Cimino JJ (2006). Heuristic evaluation of paper-based web pages: a simplified inspection usability methodology. J Biomed Inform.

[60] Wichansky AM (2000). Usability testing in 2000 and beyond. Ergonomics.

[61] Sadler GR, Lee H, Lim RS, Fullerton J (2010). Recruitment of hard-to-reach population subgroups via adaptations of the snowball sampling strategy. Nurs Health Sci.

[62] Khalil MK, Elkhider IA (2016). Applying learning theories and instructional design models for effective instruction. Adv Physiol Educ.

[63] Sandelowski M (2000). Whatever happened to qualitative description?. Res Nurs Health.

[64] Cazañas-Gordón A, de san Miguel A, Mora EP (2017). Estimating sample size for usability testing. Enfoque.

[65] Monkman H, Kushniruk A (2015). eHealth literacy issues, constructs, models, and methods for health information technology design and evaluation. Knowl Manag eLearn.

[66] (2017). UC Berkeley Library.

[67] Monkman H, Griffith J, Kushniruk AW (2015). Evidence-based heuristics for evaluating demands on ehealth literacy and usability in a mobile consumer health application. Stud Health Technol Inform.

[68] van Someren MW, Barnard YF, Sandberg JA (1994). The Think Aloud Method: A Practical Guide to Modelling Cognitive Processes.

[69] Jaspers MW, Steen T, van den Bos C, Geenen M (2004). The think aloud method: a guide to user interface design. Int J Med Inform.

[70] Irwin DE, Varni JW, Yeatts K, de Walt DA (2009). Cognitive interviewing methodology in the development of a pediatric item bank: a patient reported outcomes measurement information system (PROMIS) study. Health Qual Life Outcomes.

[71] Rubin J, Chisnell D, Spool J, Rubin J (2008). Analyze data and observations. Handbook of Usability Testing: How to Plan, Design, and Conduct Effective Tests.

[72] Brooke J, Jordan PW, Thomas B, McClelland IL, Weerdmeester B (1996). System usability scale: a quick and dirty usability scale. Usability Evaluation In Industry.

[73] Brooke J (2013). System usability scale (SUS): a retrospective. J Usability Stud.

[74] Bangor A, Kortum P, Miller J (2009). Determining what individual SUS scores mean: adding an adjective rating scale. J Usability Stud.

[75] Stinson J, McGrath P, Hodnett E, Feldman B, Duffy C, Huber A, Tucker L, Hetherington R, Tse S, Spiegel L, Campillo S, Gill N, White M (2010). Usability testing of an online self-management program for adolescents with juvenile idiopathic arthritis. J Med Internet Res.

[76] Colorafi KJ, Evans B (2016). Qualitative descriptive methods in health science research. Health Env Res Desig.

[77] Neuendorf K (2017). The Content Analysis Guidebook.

[78] Nielsen J, Landauer TK (1993). A Mathematical Model of the Finding of Usability Problems. Proceedings of the INTERACT '93 and CHI '93 Conference on Human Factors in Computing Systems.

[79] Stossel LM, Segar N, Gliatto P, Fallar R, Karani R (2012). Readability of patient education materials available at the point of care. J Gen Intern Med.

[80] Gunning R (1968). The Technique of Clear Writing.

[81] Roman LC, Ancker JS, Johnson SB, Senathirajah Y (2017). Navigation in the electronic health record: a review of the safety and usability literature. J Biomed Inform.

[82] Maramba I, Chatterjee A, Newman C (2019). Methods of usability testing in the development of eHealth applications: a scoping review. Int J Med Inform.

[83] Dear B F, Gandy M, Karin E, Ricciardi T, Fogliati V J, McDonald S, Staples L G, Perry K Nicholson, Sharpe L, Nicholas M K, Titov N (2017). The pain course: a randomised controlled trial comparing a remote-delivered chronic pain management program when provided in online and workbook formats. Pain.

[84] Chad-Friedman E, Talaei-Khoei M, Ring D, Vranceanu A (2017). First use of a brief 60-second mindfulness exercise in an orthopedic surgical practice; results from a pilot study. Arch Bone Jt Surg.

[85] Vranceanu A, Jacobs C, Lin A, Greenberg J, Funes CJ, Harris MB, Heng MM, Macklin EA, Ring D (2019). Results of a feasibility randomized controlled trial (RCT) of the Toolkit for Optimal Recovery (TOR): a live video program to prevent chronic pain in at-risk adults with orthopedic injuries. Pilot Feasibility Stud.

[86] Longmore RJ, Worrell M (2007). Do we need to challenge thoughts in cognitive behavior therapy?. Clin Psychol Rev.

[87] Hofmann SG, Gómez AF (2017). Mindfulness-based interventions for anxiety and depression. Psychiatr Clin North Am.

[88] Knoerl R, Smith EM, Weisberg J (2016). Chronic pain and cognitive behavioral therapy: an integrative review. West J Nurs Res.

[89] Goudie S, Dixon D, McMillan G, Ring D, McQueen M (2018). Is use of a psychological workbook associated with improved disabilities of the arm, shoulder and hand scores in patients with distal radius fracture?. Clin Orthop Relat Res.

[90] Rapport F, Clay-Williams R, Churruca K, Shih P, Hogden A, Braithwaite J (2018). The struggle of translating science into action: foundational concepts of implementation science. J Eval Clin Pract.

[91] Alexanders J, Anderson A, Henderson S (2015). Musculoskeletal physiotherapists' use of psychological interventions: a systematic review of therapists' perceptions and practice. Physiotherapy.

[92] Zadro J, O'Keeffe M, Maher C (2019). Do physical therapists follow evidence-based guidelines when managing musculoskeletal conditions? Systematic review. BMJ Open.

[93] Bennett S, Whitehead M, Eames S, Fleming J, Low S, Caldwell E (2016). Building capacity for knowledge translation in occupational therapy: learning through participatory action research. BMC Med Educ.

[94] Scurlock-Evans L, Upton P, Upton D (2014). Evidence-based practice in physiotherapy: a systematic review of barriers, enablers and interventions. Physiotherapy.

[95] Mauck BM, Swigler CW (2018). Evidence-based review of distal radius fractures. Orthop Clin North Am.

[96] Grivna M, Eid HO, Abu-Zidan FM (2016). Epidemiology of isolated hand injuries in the United Arab Emirates. World J Orthop.

[97] Heffernan KJ, Chang S, Maclean ST, Callegari ET, Garland SM, Reavley NJ, Varigos GA, Wark JD (2016). Guidelines and recommendations for developing interactive ehealth apps for complex messaging in health promotion. JMIR Mhealth Uhealth.

[98] Fu MR, Axelrod D, Guth AA, Wang Y, Scagliola J, Hiotis K, Rampertaap K, El-Shammaa N (2016). Usability and feasibility of health IT interventions to enhance self-care for lymphedema symptom management in breast cancer survivors. Internet Interv.

[99] Harte R, Glynn L, Rodríguez-Molinero A, Baker PM, Scharf T, Quinlan LR, ÓLaighin G (2017). A human-centered design methodology to enhance the usability, human factors, and user experience of connected health systems: a three-phase methodology. JMIR Hum Factors.

