# Supplementary material for: Development and Usability Testing of a Web-Based and Therapist-Assisted Coping Skills Program for Managing Psychosocial Problems in Individuals With Hand and Upper Limb Injuries: Mixed Methods Study
Source: JMIR Hum Factors. 2020 May 6;7(2):e17088. doi: 10.2196/17088 (PMC7240444; doi:10.2196/17088)
Supplement: Multimedia Appendix 1 [file humanfactors_v7i2e17088_app1.pdf]

Multimedia Appendix 1: Semi-structured interview guide for conducting cognitive interviews during usability testing

Now that you have used the website, we are interested in learning about what you liked and disliked about the website.

Question:

1. What did you like best about the website?
   Probes: information, layout, animations, audio quality, PowerPoint presentations, graphics, video clips, workbook etc. Can you tell more about that?
2. What did you like the least or dislike about the website?
   Probes: information, layout, animations, audio quality, PowerPoint presentations, graphics, video clips, workbook etc. Can you tell more about that?
3. How easy was to find your way around the website?
   Probes: What were the challenges of navigating through the website? What would make it easier to navigate through the site?
4. What are your thoughts on the overall look of the website?
   Probes: For example, the design, colours, and images/backgrounds, feeling warm/friendly/cold/too technical? Do you think it is visually appealing or unappealing? What would make the website more appealing? What caught your attention the first time you went through the website?
5. Tell me what you think about the information provided on the website?
   Probes: Accuracy, trustworthiness, amount of information. What needs to be added? Was the information provided helpful, clearly presented, easy to read and understand? Potential to help patients and therapists learn how to cope with psychosocial problems like depression, pain anxiety?
6. Tell me whether or not you would use HOCOS to better manage psychosocial problems?
   Probes: What would motivate you to use this website?
7. Tell me whether or not you think other therapists would be interested in using HOCOS?
   Probes: Perceive interest, recommendation friend with psychosocial issues after a HULI.
8. If you could make any changes to the website, what changes would you make?
   Probes: In terms of content, overall look, graphics?
9. Is there anything else you would like to tell us the website?
   Probes: Can you tell me more about that
