# Supplementary material for: Development and Usability Testing of a Web-Based and Therapist-Assisted Coping Skills Program for Managing Psychosocial Problems in Individuals With Hand and Upper Limb Injuries: Mixed Methods Study
Source: JMIR Hum Factors. 2020 May 6;7(2):e17088. doi: 10.2196/17088 (PMC7240444; doi:10.2196/17088)
Supplement: Multimedia Appendix 2 [file humanfactors_v7i2e17088_app2.pdf]

Multimedia Appendix 2: Heuristic Evaluation of HOCOS by ICT experts using general and readability guidelines of the Health Literacy Online (HLO) Checklist

**Domain**	**Criteria**	**HOCOS**
Domain 1. Writer actionable content	a. Identify user motivations and goals.	Yes
	b. Put the most important information first.	No – key information not consistent in the first paragraph
	c. Describe the health behavior - just the basics	Yes
	d. Stay positive. Include the benefits of taking action.	Yes
	e. Provide specific action steps.	Yes
	f. Write in plain language.	No – poor use of the active voice, use of jargon and long sentences.
	g. Check content for accuracy	Yes
Domain 2. Display Content Clearly on the Page	a. Limit paragraph size. Use bullets and short lists.	Yes
	b. Use meaningful headings.	Yes
	c. Use a readable font that's at least 16 pixels.	No – inconsistent font sizes.
	d. Use white space and avoid clutter.	Yes
	e. Keep the most important content above the fold - even on mobile.	No – key information sometimes below the fold
	f. Use links effectively.	Yes
	g. Use color or underline to identify links.	Yes
	h. Use images that help people learn.	Yes
	i. Use appropriate contrast.	Yes
	j. Make web content printer friendly.	Yes
	k. Make your site accessible to people with disabilities.	No
	l. Make websites responsive.	Yes
	m. Design mobile content to meet mobile users' needs.	No – mobile format needs a lot of editing.
Domain 3. Organize content and simplify navigation.	a. Create a simple and engaging homepage.	No – need to use links with clickable buttons. The image used does not directly reflect the planned end user.
	b. Label and organize content with your users in mind.	Yes
	c. Create linear information paths.	No – too much scrolling to get through materials.
	d. Give buttons meaningful labels.	Yes
	e. Make clickable elements recognizable.	No – users must skim across some headings before they become clickable.
	f. Make sure the browser "Back" button works.	No – inconsistent on the website
	g. Provide easy access to home and menu pages.	Yes

	h. Give users options to browse.	Yes
	i. Include a simple search function.	No – search function not available.
	j. Display search results clearly.	No – search function not available.
Domain 4. Engage Users	a. Share information through multimedia.	Yes – good use of multimedia.
	b. Design intuitive interactive graphics and tools.	No – missing interactive graphics and tools.
	c. Provide tailored information.	No – no means to tailor and guide information gathering.
	d. Create user-friendly forms and quizzes.	Yes
	e. Consider social media sharing options.	No – no social media sharing option.

Reference: US Department of Health and Human Services (DHHS), Office of Disease Prevention and Health Promotion. "Health Literacy Online: A Guide to Simplifying the User Experience." 2nd ed. 2015. Available on https://health.gov/healthliteracyonline/checklist/
