# Supplementary material for: Development and Usability Testing of a Web-Based and Therapist-Assisted Coping Skills Program for Managing Psychosocial Problems in Individuals With Hand and Upper Limb Injuries: Mixed Methods Study
Source: JMIR Hum Factors. 2020 May 6;7(2):e17088. doi: 10.2196/17088 (PMC7240444; doi:10.2196/17088)
Supplement: Multimedia Appendix 3 [file humanfactors_v7i2e17088_app3.pdf]

Heuristic	Description	HOCOS
Item 1. Immediately inform users of purpose and engage users; avoid registration.	Identify the purpose and audience on the home screen page. If unavoidable, make registration and logging in simple and obvious.	Criteria met
Item 2. Use complementary interaction methods.	Make use of alternative inputs (e.g., touch screen, barcode scanning, voice commands) and outputs (e.g., audio recordings, videos, text-to-speech engines).	Criteria met
Item 3. Leverage interactivity.	Offer interactive tools (e.g., quizzes, questionnaires, glossaries, tutorials) to engage with the information and provide performance feedback. Allow users to share information (e.g., print, e-mail) with others.	Criteria not met<br>- the little interactivity on the website does not provide participant engagement.
Item 4. Provide accurate, colloquial, comprehensive, succinct content.	Written information should be brief, relevant, and in user's vernacular.	Criteria not met<br>- poor use of plain language (grade 6 level)
Item 5. Provide tailored, flexible, layered content.	Prioritize information according to importance. If possible, personalize information. Provide succinct summaries but allow users to access more detailed information. Offer content in multiple languages.	Criteria not met<br>- content not prioritized or personalized, use of English language alone.
Item 6. Use visuals to complement text but avoid tables.	Visuals (e.g., pictures, videos, animations) may enhance written information. If unavoidable, tables should be designed as independent, simplistic representations of information.	Criteria met
Item 7. Simplistic, consistent navigation.	Keep users oriented. Use linear navigation to facilitate forward and backward movement. Use large buttons, clearly label links, and provide a search engine.	Criteria met
Item 8. Simplistic, consistent displays.	Avoid on-screen complexity. Avoid the need for scrolling by limiting information on a page/screen.	Criteria not met<br>- too much scrolling to access key information.
Item 9. Clear and comprehensive communication of risks.	Describe risk terminology in a way the users will understand. Use 100 as upper limit on bar graphs. Avoid logarithmic scales.	Criteria not met<br>- risk such as privacy/confidentiality not clearly conveyed
Item 10. Clear depiction of monitoring data and/or test results.	Emphasize values outside acceptable ranges. Facilitate pattern recognition and rapid identification of influential factors.	Not applicable
Item 11. Considerations for mobile devices.	Allow users to adjust the display size using familiar input (e.g., pinch to zoom, turning to landscape orientation). Use appropriately sized interface elements. Limit the amount of information displayed.	Criteria met

Reference: Monkman, H., J. Griffith, and A. W. Kushniruk. "Evidence-based Heuristics for Evaluating Demands on eHealth Literacy and Usability in a Mobile Consumer Health Application." Studies in Health Technology and Informatics 2015; 216:358
