# Supplementary material for: Development and Usability Testing of a Web-Based and Therapist-Assisted Coping Skills Program for Managing Psychosocial Problems in Individuals With Hand and Upper Limb Injuries: Mixed Methods Study
Source: JMIR Hum Factors. 2020 May 6;7(2):e17088. doi: 10.2196/17088 (PMC7240444; doi:10.2196/17088)
Supplement: Multimedia Appendix 4 [file humanfactors_v7i2e17088_app4.pdf]

Theme and its definition	Example of negative comments	Changes made to HOCOS website
**General comments: Comments about the overall website.**	• "In my experience, most of the therapists are not likely to go to this level of detail and understanding about it. Go for a more basic route and give them some simple tools with good examples that they could try out without having to do as much learning as this might demand of them [CH, cycle 1]"<br>• "It was feeding me information, but it did not ask me about myself. e.g. write down three key stressors in your life. Write down 5 items you can remember at 9.00 pm tonight" [AB, cycle 1]<br>• "It did not feel as engaging as I would like it to be" [RC, cycle 1]	• We broke down the module content into sections and adding a link for additional learning if desired by therapists.<br>• Included active learning in the "Stop-Pause-think" section by inviting learners to respond to questions in the PowerPoint slide.<br>• Reflective pieces were created to support learning on the website. For example, think about it for 20 seconds"
**Navigation: Comments on the ability for participants to independently move around the website, review the content and use e-tools.**	• "I think therapists might benefit from having a bit of a decision matrix/tree or a matrix…what kind of people are more likely to use this module versus another one? What's the key thing about this technique? Who's gonna Respond? What are the contraindications" [CH, cycle 2]	• Added a decision matrix on the "Introductory" page.
**Content: Comments about the materials offered by the website including PowerPoint presentations, videos, didactic information, e-tools**	• "15 mins, probably too long for mental imagery. Maybe 5 mins…Five is a very long time if you're asking people to just sit quietly and use their imagination for something. [CH, cycle 2, M1]"<br>• "I would suggest doing them together instead of the good side first and then the bad side for more active engagement for the patients…for a lot of people, visualization is pretty difficult [CH, cycle 2, M1]"<br>• "Thinking about doing an audio tape that that instead of reading alone, people can go through this in 15 min because they could just hit play on their device and actually be more present in doing activity" [TP, cycle 1, M1]<br>• "Limit the information on correlation (research) to therapists alone and not the patients" [AB, cycle 1, M3]<br>"Keep slide 34 only for the therapists" Give me a bit more scientific evidence for the sensitive brain like MRI" [AB, cycle 2, M1]<br>• "So, the see module two a, there seems to be difficult for me to, to grab because I have to remember the entire module	• Instruction on mental imagery was modified to include comments about focusing on completing the task sequence instead of focusing on how long it takes.<br>• The guideline for mental imagery was edited to suggest practicing with both hands initially.<br>• An audio recording was created to support all the activities on the website modules.<br>• References to website materials were added directly to therapist information only. Patients could find more information on the "Resource" page.<br>• We created a direct link to a section of the website that is referenced on another page of the website.<br>• We tried to follow the PowerPoint Math: 1-6-6 rule as much as possible (Have only 1 idea per slide, have at most six bullet points, Maximum 6

	before I get to this point. And so, it's quite challenging" [TS, cycle 1, M4]<br>• "This is a very condensed slide with a lot of information. Lot of wording to read. So I just find it too busy for my eyes" [TS, cycle 2, M5]	words per bullet point, this slide has 6 bullet points, Each bullet point has 6 words).
**Layout: Comments about the visual appearance of the website, including color, font size, images.**	• "I actually would like to see pain a number of times in different colors and different sizes because I think that would communicate the experience of pain is different for everybody because not everybody feels like pain as the dominant issue" [TP, cycle 2, M1]<br>• "These pictures. I think we'll be fine for therapist again for patients they are extreme. That's just from the, from the CRPS world because when people have really changed their body map, this looks threatening to them" [IN, cycle 2, M1]<br>• "I would change running to walking. Because all these three are pretty high level. You know, you're probably talking to people who aren't at this level" [AB, cycle 2, M3]<br>"Sorry, the exercise part, you may not want to have a jog or a walk or something. Something softer and gentler" [VF, cycle 2, M3]<br>• "It looks like the picture is someone in white coats counseling someone in pink cloth. Um, so it doesn't reflect much about the musculoskeletal injury, distressing symptoms such as pain" [TS, cycle 1, M1]<br>• "Looks like down time is all about sports in this slide, which is not necessarily so depending on who the person is. So pictures there could include reading a book or doing some gardening, that sort of thing. In any case, make sure it's not just sports that are included in this slide. Give people more ideas. [RC, cycle 2, M5]	• We introduced the concept of coloring pain.<br>• Modified the pictures on the website to ensure they are sensitive to the needs of potential users on the website.<br>• Reviewed the pictures used to reflect different activity suggested on the website.<br>• Reviewed the pictures on each page to reflect the information on each slide.<br>• We ensured a proper mix of IADLS, ADLS, Occupations and sporting activities on the website.
**Design: Comments on the functionality of the website, including graphic design and content production.**	• "Make sure that it's going to display well on other devices like the smart phone which many younger adults use for everything" [CH, cycle 2].<br>• "So, I think finding something that makes a lot of sense to them already that they don't need to do a lot of new learning in the intro piece to the module is important" [CH, cycle 1, M1]	• Edited the content to fit the pages of hand-held devices.<br>• Decided against make Mental Imagery the first activity in Module 1.<br>• Introduced the Injustice Experience Questionnaire to assess perceived injustice.

	• "You need to have a measure on injustice because the fractured relationship is sometimes with the employer, or the co-workers or with WSIB" [CH, cycle 1, M1]<br>• "Start with the thinking traps… Because that's really developing awareness is the first part of that. Right? Before you can do any solving of anything" [CH, cycle 2]<br>• "So, in addition to teaching them what to do and how to do it, it's important to help them plan out how to be compliant. [CH, cycle 2]"<br>• "I think sleep should be a stand-alone model because it's a problem that uh, sometimes it's related to pain or sometimes it's related to depression, sometimes related to anxiety, anxiety, stress can be many things and people" [CH, cycle 1]<br>• "Consider doing PART A, B and C over 1-week period. So, then you could do a week because then someone can do 10 minutes and they're okay to and then the next day do 10 minutes." [PP, cycle 1, M3]<br>• "Create a summary page for key concepts for take home messages" [JT, cycle 1, M4]	• In Module 2, we introduced the thinking traps before discussing the cognitive restructuring.<br>• Incorporate a 'how to make this work' section.<br>• Sleep education was included in the resource section.<br>• We broke down the PowerPoint presentation into sections to reduce viewer fatigue.<br>• We added a "summary page" at the end and beginning of every module"
**Language: Comments on the grammar, word choice, header titles and**	• "The sentences are too long, but it's still. That's a little bit text dense. I'm worn just from the first part. That first paragraph. So, I might, I might even break that into a second pair [PP, cycle 1]<br>• "Just be careful. The no pain, no gain. I know it's a play on words here, but that's an old myth that drives people to overwork. So, knowing pain equals knowing gain, maybe" [AB, cycle 2, M1]<br>• "Saying husband sounds too gendered. [JT, cycle 2, M4]<br>• "I think I would use language that like if you were to maybe if you think about it in the sense of, you know, I'm talking to a sixth grader, how am I going to speak to them and then therefore how can it be worded because your content is good" [JT, cycle 1, M4]<br>• "Some terms are too big. Make the terms more related to day to day life here will be better. The materials are also trying to help clinicians talk to clients. So, being able to use simple words in the presentation is training the clinicians to	• We made the sentences shorter with smaller paragraphs.<br>• Edited the content to ensure slogans were not misleading.<br>• Preferable to say spousal partner"<br>• Ensured that the patient documents and materials were at Grade 6 level.<br>• Reviewed the words to find synonyms that would enhance lay man understanding.<br>• More clarification was provided to support the metaphors used.

	come up with a grade five language to talk to clients" [TS, cycle 2, M5]<br>• "Okay. I understand that you want to do metaphors. Metaphors need explanation" [VF, cycle 2, M5]	
